# Rare crystalline nephropathy leading to acute graft dysfunction: a case report

**DOI:** 10.1186/s12882-019-1616-3

**Published:** 2019-11-21

**Authors:** Sahil Bagai, Dinesh Khullar, Bhavna Bansal

**Affiliations:** 1 Department of Nephrology and Renal transplant medicine, Max Superspeciality Hospital, 1 & 2, Press Enclave Marg Saket, New Delhi, Delhi, 110017 India; 2 Department of Pathology, Max Superspeciality Hospital, 1 & 2, Press Enclave Marg Saket, New Delhi, Delhi, 110017 India

**Keywords:** APRT, Crystalline nephropathy, Dihydroxyadenine

## Abstract

**Background:**

Adenine phosphoribosyl transferase (APRT) deficiency is a rare genetic form of kidney stones and/or kidney failure characterized by intratubular precipitation of 2,8 dihydroxyadenine crystals. Early diagnosis and prompt management can completely reverse the kidney injury.

**Case presentation:**

44 year old Indian male, renal transplant recipient got admitted with acute graft dysfunction. Graft biopsy showed light brown refractile intratubular crystals with surrounding giant cell reaction, consistent with APRT deficiency. Patient improved after receiving allopurinol and hydration.

**Conclusion:**

APRT forms a reversible cause of crystalline nephropathy. High index of suspicion is required for the correct diagnosis as timely diagnosis has therapeutic implications.

## Background

Adenine phosphoribosyltransferase (APRT) deficiency is a rare autosomal recessive disease in the purine salvage pathway that causes excessive production of 2,8-dihydroxyadenine (DHA). APRT is an enzyme that converts adenine into adenosine monophosphate. Its deficiency results in the accumulation of adenine, which in turn gets converted to 2,8-dihydroxyadenine (DHA) by xanthine oxidase. DHA forms insoluble crystals in urine. Renal involvement occurs in isolation because DHA is not systemically deposited [1]. It manifests as nephrolithiasis or crystalline nephropathy in homozygous patients [2]. Diagnosis is suggested by either detection of urinary 2,8-DHA crystals or histologic findings of crystal nephropathy or kidney stone analysis. The disease should be confirmed by genetic testing or demonstration of absence to APRT activity in red blood cell lysate assay [3].

Most common cause of crystalline nephropathy seen in post transplant setting in developing world is primary hyperoxaluria which is a close mimicker of APRT deficiency. Inappropriate diagnosis can have serious therapeutic implications as former requires combined liver kidney transplant and latter requires only medical management.

We report a case of renal transplant recipient who had no past history of nephrolithiasis but developed APRT related nephropathy in immediate post transplant period and successfully recovered after treatment.

## Case presentation

**Fig. 1:**
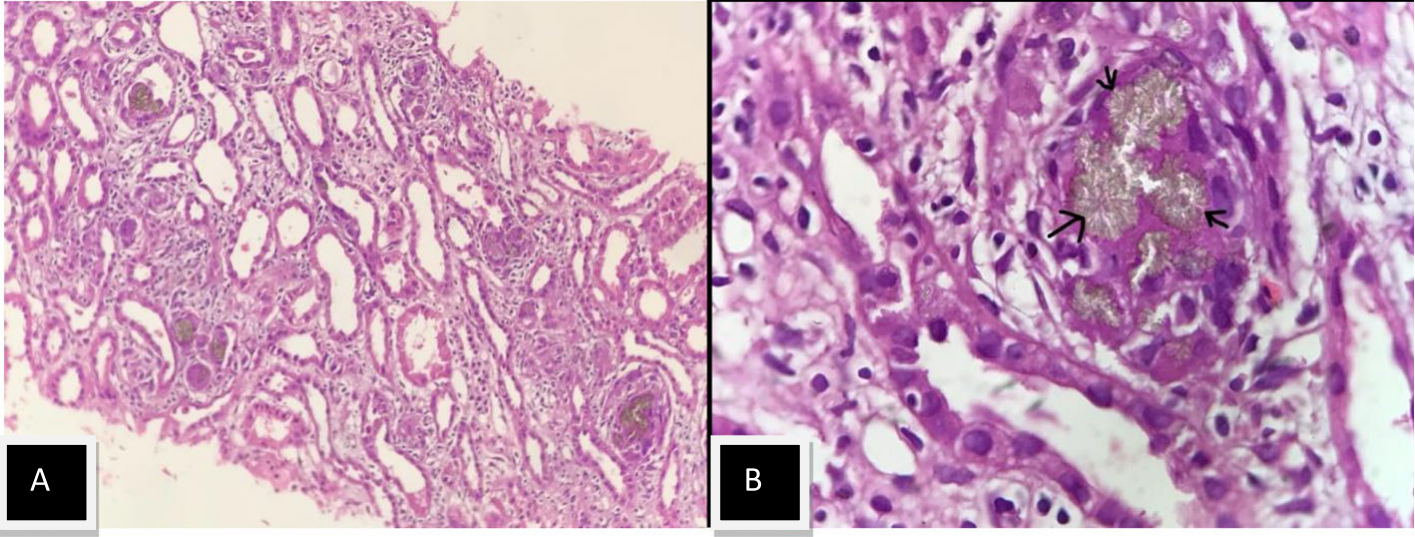
Renal biopsy microphotograph showing multiple light brown intra tubular crystals with surrounding giant cell reaction. [PAS stain 10X(Fig. 1a) and 40x(Fig. 1b)]

**Fig. 2:**
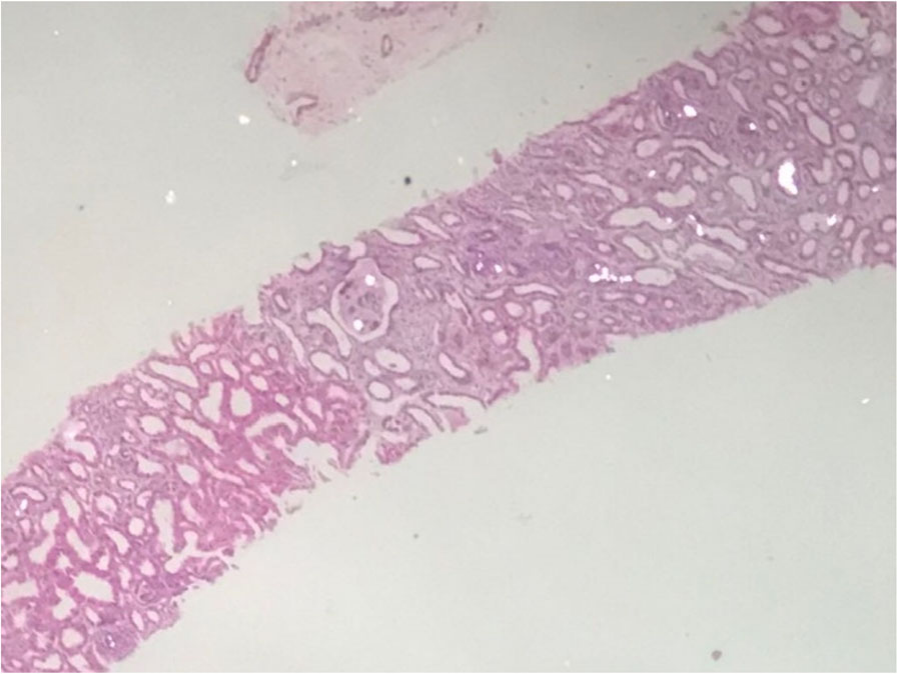
Renal biopsy microphotograph showing multiple birefringent crystals under polarized light

44 year old Indian male was diagnosed as having chronic kidney disease in 2015, basic disease unknown, not evaluated in detail although he had family history of renal disease. He reached end stage renal disease status in December 2016 and was initiated on hemodialysis via a tunneled catheter and maintained on thrice/week dialysis. He underwent live donor renal allograft transplantation with wife as the donor in March 2017 at a private hospital, Delhi. He received Basiliximab as induction and was maintained on tacrolimus, mycophenolate mofetil (MMF) and steroids. His immediate post transplant period was uneventful apart from one episode of urinary tract infection which was managed with antibiotics. His serum creatinine was around 1.0–1.1 mg/dl. Two months post transplantion he presented to our hospital with asymptomatic rise in serum creatinine to 2.0 mg/dl. Patient denied any history of non compliance. Physical examination was essentially normal. Routine investigations showed hemoglobin 10.5 g/dl, total leucocyte count 5*10 [3]/cumm and platelet count were 2.95*10 [4]/cumm, urine routine showed protein 1+, few RBC’s and no pus cells. Urine protein creatinine ratio was 0.48 and tacrolimus level was 9.6 ng/ml. Graft kidney doppler was normal. In view of unexplained acute graft dysfunction, graft biopsy was done which showed normal glomeruli with presence of multiple light brown annular intratubular crystals with surrounding giant cell reaction on light microscopy. These crystals were refractile under polarizing microscope. Immunofluorescence microscopy was negative. (Fig. 1 A, Fig. 1B and 2). Patient had no past history of nephrolithiasis but had a strong family history of nephrolithiasis. His maternal cousin and elder brother had died of kidney disease. He was worked up for primary hyperoxaluria in view of suspicion on kidney biopsy but normal plasma oxalate (3.34 g/1.73m^2^/day) and 24 h urinary oxalate levels (55.56 mg) ruled out the possibility. To ascertain the cause genetic analysis was done which revealed a novel non sense varation in the exon 3 of the *APRT* gene that resulted in stop codon and premature truncation of the protein at codon 87 s/o *APRT* deficiency. The patient was advised good hydration and low purine diet and started on allopurinol 200 mg daily. He attained a baseline serum creatinine within 1 month of therapy. His Uric acid level before initiation of therapy was 7 mg/dl and maintained between 5 and 6 mg/dl on therapy.

## Discussion and conclusion

APRT deficiency is a rare inborn error of purine metabolism caused by mutation in the APRT gene. It is characterized by the presence of 2,8 DHA crystals which are radiolucent and are often confused with other xanthine crystals. Most cases of DHA accumulation in urine have been reported primarily from Japan, France, Unites states of America and Iceland. Four cases of APRT deficiency have been published from Indian subcontinent but none showed crystalline nephropathy in renal transplant recipient [4–6]. Nephrolithiasis and crystalline nephropathy are two ways in which kidneys are affected [1]. Recurrent nephrolithiasis is a far more common presentation than crystalline nephropathy. It is usually misdiagnosed and mistreated as uric acid/xanthine stones as both are radiolucent stones and are not easily differentiated by stone analysis in patients presenting with nephrolithiasis [7]. Post renal transplantation, pathologists might mistake DHA disease as primary hyperoxaluria if they are not careful. The DHA crystals are brownish green as against oxalate crystals which are transparent. The APRT gene located on 16q24 is 2.6 kb long with five exons. Diagnosis is suggested by either detection of urinary 2,8-DHA crystals or histologic findings of crystal nephropathy or kidney stone analysis. The disease should be confirmed by genetic testing or demonstration of absence to APRT activity in RBC lysate [8]. APRT deficiency presents as Type I (complete deficiency in vivo and in vitro) and Type II (complete deficiency in vivo with 10–25% activity in vitro) [9].

In our patient we had suspected the disease entity keeping in mind the strong family history of renal disease and by the presence of polarized intra tubular crystals on biopsy with normal plasma and urinary oxalate levels. Since APRT is a close mimicker, the final confirmation in our case was established with the help of genetic study which revealed a novel non sense mutation in exon 3.

Managing APRT deficient patients is rather straight forward. Xanthine oxidase inhibitor, low purine diet and good hydration form the pillars for management [10]. Time to diagnosis is the corner stone in case of crystalline nephropathy as late diagnosis would still lead to graft loss [11]. This is similar to a French study wherein only 1 patient out of 6 achieved good graft function despite correct diagnosis [12]. Fortunately our patient presented early and we could successfully manage him with good hydration, low purine diet and allopurinol.

Learning points from our case:

- Early recognition is critical as late diagnosis would lead to a progressive disease
- Intratubular crystallization on biopsy with negative work up for hyperoxaluria warrants a need for APRT evaluation.
- Life time xanthine oxidase inhibitor, low purine diet and high fluid intake forms the backbone of treatment in these patients.
- DHA crystals in urine or in biopsy are suggestive of APRT deficiency. However, confirmation is done with help of RBC enzyme assay or genetic testing.

## Data Availability

Not applicable

## Abbreviations

2,8-DHA: 2,8-dihydroxyadenine crystals
APRT: Adenine phosphoribosyltransferase
MMF: Mycophenolate mofetil
